# Regulation of Smooth Muscle Cell Proliferation by NADPH Oxidases in Pulmonary Hypertension

**DOI:** 10.3390/antiox8030056

**Published:** 2019-03-05

**Authors:** John C. Huetsch, Karthik Suresh, Larissa A. Shimoda

**Affiliations:** Department of Medicine, Division of Pulmonary and Critical Care Medicine, Johns Hopkins School of Medicine, Baltimore, MD 21224, USA; jhuetsc1@jhmi.edu (J.C.H.); ksuresh2@jhmi.edu (K.S.)

**Keywords:** NADPH oxidase, pulmonary arterial smooth muscle, reactive oxygen species

## Abstract

Hyperproliferation of pulmonary arterial smooth muscle cells is a key component of vascular remodeling in the setting of pulmonary hypertension (PH). Numerous studies have explored factors governing the changes in smooth muscle cell phenotype that lead to the increased wall thickness, and have identified various potential candidates. A role for reactive oxygen species (ROS) has been well documented in PH. ROS can be generated from a variety of sources, including mitochondria, uncoupled nitric oxide synthase, xanthine oxidase, and reduced nicotinamide adenine dinucleotide phosphate (NADPH) oxidase. In this article, we will review recent data supporting a role for ROS generated from NADPH oxidases in promoting pulmonary arterial smooth muscle cell proliferation during PH.

## 1. Introduction

Arising from various etiologies, pulmonary hypertension (PH) is a complicated condition diagnosed by increased pulmonary arterial pressure. Historically, the threshold for diagnosis has been a mean pulmonary arterial pressure of ≥25 mm Hg, although recently, reductions to >20 mmHg when combined with other hemodynamic abnormalities have been proposed [1]. The increase in pulmonary arterial pressure is due to both contraction and remodeling of the pulmonary vessels. The World Health Organization has clinically classified PH into five main groups (Table 1), based on the hemodynamics, underlying cause, clinical presentation, and therapeutic responsiveness [1]. Perhaps because of this complexity, our understanding of the mechanisms underlying disease development and progression remains incomplete, and treatment options are limited to targeting a few main pathways primarily involving the reduction of vasomotor tone. However, significant vascular remodeling is a component of all forms of PH, although the relative contribution of remodeling and contraction may vary. Thus, the development of treatments aiming to de-remodel the pulmonary vasculature would be beneficial, but requires a better understanding of the processes involved.

Histological analysis provided early evidence of the changes in the pulmonary vascular wall components that occur during the development of PH. Hyperproliferation of smooth muscle, fibroblasts, and endothelial cells (ECs) is evident, as is the migration of muscle into typically non-muscular small arterioles. Numerous studies have explored factors governing the changes in cell phenotype that lead to the increased wall thickness, and have identified various potential candidates. For example, a role for reactive oxygen species (ROS) has been well documented in PH (reviewed in [2,3,4,5]). ROS can be generated from a variety of sources, including mitochondria, uncoupled nitric oxide synthase, xanthine oxidase, and reduced nicotinamide adenine dinucleotide phosphate (NADPH) oxidase (Nox) (reviewed in [6,7,8]), and are important for the control of a variety of cell functions. In vascular smooth muscle cells (VSMCs), processes modulated by ROS include calcium homeostasis, transcriptional regulation, response to hypoxia, and activation of apoptotic pathways [9]. In this article, we will review recent data supporting a role for ROS generated from NADPH oxidases in promoting pulmonary arterial smooth muscle cell proliferation during PH.

## 2. NADPH Oxidases

Fundamentally, ROS generation utilizes electron transport from a donor (i.e., reduced nicotinamide adenine dinucleotide (NADH), reduced flavin adenine dinucleotide (FADH2), NADPH) via an intermediate carrier (i.e., a heme-containing protein, cytochrome, or ubiquinol) to molecular oxygen, generating superoxide radicals. The classic example of this reaction occurs in mitochondria, where electron carriers, NADH and FADH2, donate electrons, which are carried via ubiquinol, cytochrome c, and other intermediates through the electron transport chain, terminating in the donation of electrons to molecular O_2_. On a smaller scale, the same process occurs in other cellular compartments. In the cytosol, a major executioner of this ROS producing reaction is the family of Nox enzymes.

The earliest described Nox [10], Nox2, was discovered in the context of hydrogen peroxide (H_2_O_2_) production in neutrophils (i.e., “respiratory burst” [11]). Deficiency of Nox2-mediated ROS generation in phagocytes leads to chronic granulomatous disease (CGD) in humans. However, Nox expression is near-ubiquitous, and Nox isoforms are present in virtually every tissue bed, functioning to produce both basal and stimulus-induced ROS.

A variety of Nox isoforms exist. As mentioned earlier, Nox2 has been best characterized in phagocytes, but has also recently been found in VSMCs. Nox4 is by far the best characterized Nox isoform in non-immune cells, and has been extensively studied in cancer. More recently, Nox5, and a distinct subfamily of Noxs termed dual oxidase (Duox1-2), have also been described. For the purposes of this review, discussion will be limited to Nox1-4, as these have been the most extensively studied in VSMCs. As discussed below, Noxs share several isoform-independent common features including: Mechanism of ROS generation, requirement of binding partners for activation, and modification of activity based on subcellular compartmentalization.

To make ROS, Nox must first receive electrons from NADPH. While Noxs can also receive electrons from NADH, this process is thought to be less efficient [12]. To initiate this process, conserved NADPH and flavin adenine dinucleotide (FAD) binding domains at the C-terminal tail facilitate electron transfer from NADPH to FAD. Mutations in the portion of the binding domain that facilitates NADPH-FAD electron transfer do not change Nox2 levels or localization, but clinically cause chronic granulomatosis disease [13], suggesting that this motif is critical for Nox2-mediated ROS generation in neutrophils. Next, electrons are transferred to heme centers within the main Nox protein. Similar to what occurs in complex III in the mitochondria, the electron transfer at this step has to be made in a step-wise fashion (i.e., one electron a time) due to energetic considerations and the inability of the heme center to accept more than one electron at a time. Thus, over a series of two reactions, electrons from FADH2 are transferred to heme groups within Nox. However, unlike the mitochondria, where step-wise electron delivery is ultimately processed and coordinated in complex IV to reduce O_2_ to water, in the case of Nox, single electron transfer to heme continues onward one electron at a time, from heme to molecular O_2_, producing superoxide [14]. Of note, certain isoforms, such as Nox4, have been reported to generate H_2_O_2_, rather than superoxide, as the major radical in response to activation [15]. However, as discussed in detail elsewhere [8], this likely reflects the close association of dismutases with Nox4 in various cell types, rather than a fundamental difference in how ROS production is executed. The species of ROS generated by Nox may have important functional consequences. Superoxide can react with nitric oxide (NO), resulting in the depletion of this vasodilator as well as the generation of peroxynitrite, which functions as an oxidant and a nitrating agent, and ultimately drives the formation of more superoxide via an uncoupling of NO synthase [16]. H_2_O_2_, in contrast, does not deplete NO and indeed may aid NO generation via activation of NO synthase [16]. Despite these differences in the type of ROS generated, O_2_ is the primary destination of electron transfer by Nox, regardless of the isoform [17]. Although all the individual electron carrier components described above are present within the main Nox enzyme, electron transfer does not occur without the binding of critical subunits, as described below.

**Figure 1:**
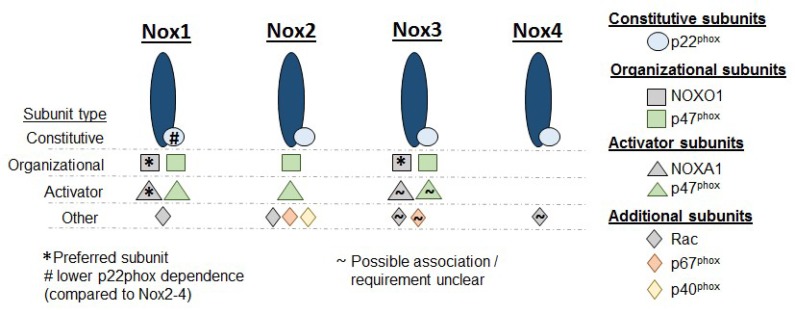
Nox isoforms and subunits. Diagram showing subunit requirements for the isoforms 1–4 of NADPH oxidase (Nox). In addition to the base Nox enzyme, p22^phox^ is constitutively required across Nox1–4. While Nox4 does not require any further subunits before ROS generation can commence, Nox1–3 require additional organizational and activator subunits. NOXO1 = NADPH oxidase organizer 1; NOXA1 = NADPH oxidase activator 1.

Proper activation of Nox requires isoform-specific binding partners. Details regarding the specific binding partners required for each Nox isoform by tissue and stimulus are extensively described in previous reviews [8,18,19]. In general, Nox subunits can be grouped in three categories: (1) The constitutive subunit, p22^phox^; (2) the organizational subunit, p47^phox^; and (3) all other subunits. p22^phox^ is constitutively required for functioning across Nox1-4. In some isoforms, such as Nox2, the stability of the Nox protein itself is p22^phox^-dependent [20,21]. Nox4 has the lowest subunit requirement; association with p22^phox^ alone is sufficient for proper function. However, for the other Nox isoforms, p47^phox^ interacts with p22^phox^ and serves as a link between p22^phox^ and multiple other recruited subunits, such as p40^phox^ and Rac [22]. p47^phox^ has, thus, been described as an “adaptor” or “organizer” subunit. Convergence of p22^phox^, p47^phox^, and additional subunits onto Nox completes the assembly of the Nox complex, and ROS production is initiated in the subcellular domain where the assembly process has occurred. There is great heterogeneity in the subunit requirements across Nox isoforms and some subunits can serve as adaptors for multiple Noxs (Figure 1).

Lastly, Noxs are subject to significant subcellular targeting. The specific subcellular destination of each Nox can be tissue specific. In phagocytes, Nox2 is sequestered in granules, which fuse to the cell membrane during respiratory bursts. Other sites, such as the perinuclear space and the neuronal synapse, have also been described as targets of Nox delivery. In the case of Nox4, localization to focal adhesions [23] and, interestingly, the nucleus [24] has been observed. Despite these and other observations, the details of whether the movement of Nox to a specific subcellular domain changes its resting or inducible activity remains under investigation.

### 2.1. Noxs and the Regulation of Smooth Muscle Proliferation in Pulmonary Hypertension

While the role of Noxs has been extensively studied in a variety of diseases, including cancer, chronic granulomatous disease, infections, and a spectrum of fibrotic diseases, including idiopathic pulmonary fibrosis (IPF) [25], exploration of Nox isoform expression and function in PAH has been relatively limited, as detailed below.

#### 2.1.1. Nox1

Nox1 is expressed in pulmonary arterial smooth muscle cells (PASMCs), with increased Nox1 expression found in PAs from monocrotaline (MCT)-treated rats [26,27] and PASMCs from PAH patients [28]. Nox1 expression is unchanged in PAs from other animal models of PH, including the Sugen-hypoxia rat, Fawn-Hooded rat, or chronically-hypoxic mouse [27].

Global constitutive Nox1 deficiency in male mice results in the spontaneous development of PH at room air over a period of 18 weeks [29]. These mice exhibit medial hypertrophy in the pulmonary vasculature, and PASMCs isolated from them had higher cell numbers in culture. Interestingly, the increased cell number was not due to increased proliferation, but rather was associated with reduced apoptosis (i.e., less cell turnover), resulting from decreased K_v_1.5 protein expression and increased intracellular potassium levels. Notably, in the Nox1-deficient mice, no compensatory changes in Nox2 or Nox4 expression were noted. In stark contrast, global constitutive Nox1 deficiency in female mice at room air does not result in PH, and is protective from the development of PH and pulmonary vascular remodeling in the setting of sustained chronic hypoxia (CH) [28], highlighting the importance of sex hormones in the pulmonary vasculature. Consistent with Nox1 promoting PH in females, the estrogen metabolite, 16βOHE1, upregulates Nox1 expression and increases ROS production and proliferation in human PASMCs in a Nox1-dependent manner [28].

Nox1 appears to serve as an important node in the control of human PASMC function, as it not only mediates the effect of estrogen, but also of serotonin upon PASMC proliferation. In vitro, serotonin increases Nox1 expression in human PASMCs, which is necessary for augmented ROS production, proliferation, and generation of the profibrotic proteins, matrix metalloproteinase 2 and 9 (MMP2 and MMP9) [30]. All of these changes would be expected to promote the development of vascular remodeling in PH.

Nox1 is further implicated in the pathogenesis of the rat MCT model of PH [26]. PASMCs isolated from rats with MCT-induced PH exhibit increased Nox1 expression, which is necessary for increased intracellular superoxide levels and enhanced proliferation and migration. Notably, in contrast to the results observed in mice, in this model, Nox1 knockdown had no effect on PASMC apoptosis. Taken together, the effects of Nox1 on PASMC function and pulmonary vascular remodeling appear to be highly dependent upon both species and sex.

#### 2.1.2. Nox2

Nox2, often referred to as phagocyte NAPDH oxidase, plays a well characterized role in antimicrobial host defense via the generation of ROS by neutrophils, eosinophils, and macrophages. Composed of the membrane-bound subunits, gp91^phox^ (often referred to simply as Nox2) and p22^phox^, Nox2 is known to be well expressed in phagocytes. There is some contention, however, as to the expression of Nox2 in the pulmonary vessel wall. Archer et al. [31] characterized gp91^phox^ expression in the media and p22^phox^ expression in the endothelium of mouse resistance pulmonary arteries, although both were more abundant in alveolar macrophages and airway epithelium. Others corroborated the presence of gp91^phox^ in mouse [32] and rat [27,33] pulmonary arteries, and gp91^phox^ expression has been documented in pig PASMCs and pulmonary arterial endothelial cells (PAECs) [34]. In some studies, however, gp91^phox^ expression was not detected in rat PASMCs [26] or in mouse pulmonary arteries [35].

Changes in Nox2 expression have been interrogated in several models of PH. While increases in gp91^phox^ were reported in the MCT rat model [27,33], other studies found no change in gp91^phox^ expression in mice exposed to CH [32], chronic intermittent hypoxia (CIH) [36], or MCT [37], rats exposed to MCT [26], or the Fawn-Hooded rat [27], a model that spontaneously develops PH. The reasons for these discrepancies are not completely clear, but may be related to the method of measuring expression or to the size/location of the vessels being analyzed, and for in vivo studies, the expression of Nox2 by nearby adherent macrophages may confound attempts to measure its expression in vascular wall cells.

While data detailing any potential direct effect of Nox2 upon PASMC remodeling remain sparse, there has been some limited exploration of the effect of genetic deletion of Nox2 in models of PH. In the CH mouse, gp91^phox^ deficiency is protective against the development of medial wall thickening in distal pulmonary arteries and against elevations in right ventricular pressure; the knockout mice lack CH-induced increases in pulmonary artery superoxide levels seen in wild-type mice [32]. Similar findings are noted in a CIH mouse model, in which the loss of gp91^phox^ results in attenuated pulmonary vascular remodeling and right ventricular hypertrophy (RVH) [36]. In this study, gp91^phox^-deficient mice were spared from CIH-induced increases in whole lung protein kinase B (Akt) and platelet-derived growth factor receptor beta (PDGRβ) phosphorylation, suggesting that Nox2 is important in driving proliferative signaling pathways. As these studies utilized global constitutive knockouts, it remains unclear whether Nox2 activity in phagocytic cells or PAECs/PASMCs, or both, is necessary for hypoxia-induced vascular remodeling. Further, Nox4 expression is increased in the wildtype CIH mouse lung, but not in the gp91^phox^ knockout [36], raising the possibility that any observed effects of Nox2 on pulmonary vascular remodeling may have been mediated by Nox4.

#### 2.1.3. Nox4

By contrast, the literature on Nox4 and PASMC function is considerably more extensive. Numerous studies have documented Nox4 mRNA and protein expression in vitro in PASMCs, but there remains some discrepancy regarding the localization of Nox4 expression in vivo. Staining of small diameter mouse pulmonary arteries as well as sections from both healthy and PAH patient lungs revealed Nox4 expression predominantly in the media [38]. However, in another study, staining of both rat and human pulmonary arteries found Nox4 was present in the endothelium and adventitia, but absent in the media [27]. Staining of human control and IPF lung sections also revealed Nox4 staining primarily in fibroblasts and endothelial cells (along with epithelial cells) [39]. In the systemic vasculature, Nox4 expression has been found to be greater in ECs and fibroblasts than in SMCs [40,41]. In sum, there is evidence that Nox4 is expressed in all vascular cell types, with disparate reports of expression levels potentially reflecting differences in technique, species, and exposure/disease-state. In both animal models of PH and PH patients, Nox4 expression is consistently increased, including in pulmonary arteries [38] or lungs [42] from mice exposed to CH or CIH [36], in pulmonary arteries from Fawn-Hooded rats or rats exposed to CH, MCT, or a combination of SU5416 and CH [27,33,43], in pulmonary arteries and PASMCs from a lamb model of persistent pulmonary hypertension of the newborn (PPHN) [44], and in pulmonary arteries [38] and PASMCs [28,43] from patients with PH. Additionally, genomic studies have found that genetic variation in Nox4 is associated with portopulmonary hypertension [45].

**Figure 2:**
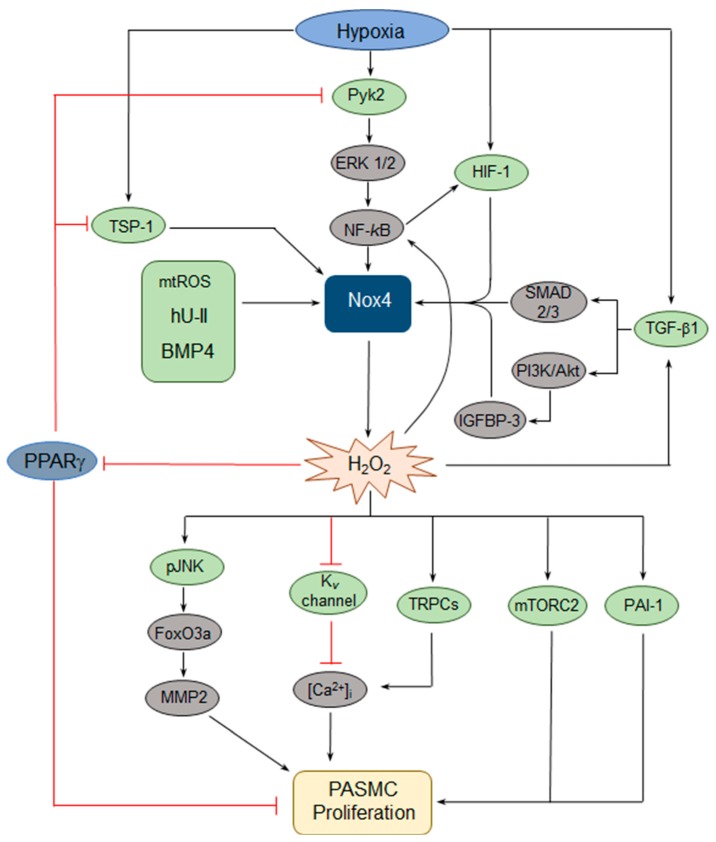
Schematic of key pathways involved in Nox4-mediated effects on pulmonary arterial smooth muscle cell (PASMC) proliferation. Hypoxia upregulates Nox4 via several pathways, including a proline-rich tyrosine kinase 2 (Pyk2)-extracellular signal-regulated kinase (ERK 1/2)-nuclear factor-kappa B (NF-kB) axis, hypoxia-inducible transcription factor 1 (HIF-1), transforming growth factor β1 (TGF-β1), and thrombospondin-1 (TSP-1). Other activators of Nox4 include human urotensin II (hU-II), bone morphogenetic protein 4 (BMP4), and mitochondrial reactive oxygen species (mtROS). Importantly, several of these pathways include feed-forward loops, some of which feature peroxisome proliferator-activated receptor γ (PPARγ) as a key component. Downstream effectors of Nox4-derived hydrogen peroxide (H_2_O_2_) include phosphorylated c-Jun-NH(2)-terminal kinase (pJNK), mammalian target of rapamycin 2 (mTORC2), plasminogen activator inhibitor-1 (PAI-1), and multiple pathways that increase intracellular calcium ([Ca^2+^]_i_). PI3K = phosphatidylinositol 3-kinase; Akt = protein kinase B; IGFBP-3 = insulin-like growth factor binding protein-3; FoxO3a = Forkhead Box O3a; MMP2 = matrix metalloproteinase 2; K_v_ = voltage-gated potassium; TRPC = transient receptor potential channel.

Given that Nox4 expression is upregulated in models of PH and that dysregulated ROS contributes to PASMC dysfunction, the upstream signaling pathways that activate Nox4 and downstream pathways that lead to changes in PASMC behavior have been of interest. Hypoxia has been found to consistently upregulate Nox4 expression in PASMCs and, in a series of papers [46,47,48,49,50], both upstream regulators and downstream effectors of hypoxic Nox4 induction have been identified (Figure 2). Hypoxia activates the proliferation of human PASMCs in a Nox4-dependent manner [50]. Hypoxic induction of Nox4 occurs via a pathway involving, sequentially, proline-rich tyrosine kinase 2 (Pyk2) activation, extracellular signal-regulated kinase (ERK) 1/2 activation, and nuclear factor-kappa B (NF-κB) activation, which binds to the Nox4 promoter [46,50]. In turn, Nox4 upregulation results in increased H_2_O_2_ production and a reduction of peroxisome proliferator-activated receptor gamma (PPARγ) expression and activity, which leads to increased proliferation [49]. Importantly, the loss of PPARγ further activates the Pyk2→ERK1/2→NF-κB→Nox4 axis, creating a feed-forward loop resulting in further generation of H_2_O_2_ [46,48]. PPARγ also negatively regulates Nox4 via a different pathway, by blocking hypoxic induction of thrombospondin-1 (TSP-1) expression, which in turn upregulates Nox4 [47]. Others have identified NF-κB variably as an upstream regulator [51] or a downstream effector [44,52] of Nox4 upregulation, providing further evidence for a NF-κB→Nox4 positive feedback loop.

Additional pathways involved in hypoxic upregulation of Nox4 have been defined, including the hypoxia-inducible transcription factors (HIFs). In human PASMCs, hypoxia induces HIF-1α, resulting in the HIF-1 transcriptional complex (composed of HIF-1α and β subunits) binding to the Nox4 promoter at a hypoxia-response element (HRE), leading to Nox4-dependent increases in proliferation and migration [53]. There also appears to be a Nox4→HIF feed-forward loop in human PASMCs, as Nox4-induced ROS upregulate HIF-1α transcription via activation of NF-κB, which binds to a site in the HIF-1α promoter [52]. Additionally, Nox4 upregulation increases HIF-2α protein levels by decreasing hydroxylation of HIF-2α and thereby attenuating von Hippel-Lindau (VHL)-mediated degradation [54]. Whether HIF-2α in turn modulates Nox4 expression remains unclear.

Transforming growth factor (TGF)-β1 has also been shown to function upstream and downstream of Nox4, suggesting yet another feed-forward loop. In normoxia, TGF-β1-induced PASMC proliferation requires the upregulation of Nox4 expression via Smad 2/3 [55]. In hypoxia, TGF-β1 has also been found to induce Nox4 expression and human PASMC proliferation through another pathway, involving activation of the phosphatidylinositol 3-kinase (PI3K)/Akt pathway and subsequent increased expression of insulin-like growth factor binding protein-3 (IGFBP-3) [56]. In vivo pharmacologic inhibition of Nox4 prevents the hypoxic induction of TGF-β1 in mouse lung homogenates, suggesting that Nox4 can in turn regulate TGF-β1 [57].

Human urotensin II (hU-II), a peptide known to serve as a potent vasoconstrictor, has been shown to increase human PASMC proliferation through several Nox4-dependent pathways. First, the hU-II-induced increase in Nox4 expression results in the activation of both mitogen-activated protein kinases (MAPKs) and the PI3K/Akt pathway, with a subsequent increase in plasminogen activator inhibitor-1 (PAI-1) expression and PASMC proliferation [58]. Second, hU-II-induced Nox4 expression also results in c-Jun-NH(2)-terminal kinase (JNK) phosphorylation, resulting in the activation of the Forkhead Box O3a (FoxO3a) transcription factor (via dissociation from 14-3-3), and subsequent increased expression of MMP2, ultimately leading to increased PASMC proliferation [59].

Other pathways regulating Nox4 expression in PASMCs have also been identified. For example, cyclic stretch of PASMCs from fetal lambs, thought to mimic pulsatile distension of the vascular wall at increased pulmonary arterial pressures, increased Nox4 expression with a subsequent increase in cyclin D1 expression, a factor important in the control of proliferation [51]. In this study, Nox4 upregulation was dependent upon mitochondrial complex III-induced activation of NF-κB, suggesting that mitochondrial-derived ROS are relevant to Nox4 induction. Similarly, hypoxic induction of mitochondrial H_2_O_2_ increases Nox4 expression [60]. While this work was done in PAECs, together these studies implicate mitochondrial dysfunction in Nox4 upregulation. Finally, in mice genetically deficient for toll-like receptor 4 (TLR4), PH develops spontaneously; PASMCs from TLR4-deficient mice exhibit increased Nox1 and Nox4 expression, suggesting that TLR4 negatively regulates Nox [61].

There has also been elucidation of a handful of further effector pathways downstream of Nox. In rat PASMCs, Nox4 activation is necessary for hypoxia-induced reduction of K_v_ currents, with Nox4-mediated oxidation of the K_v_1.5 channel associated with decreased potassium current [62]. K_v_ channel inhibition leads to membrane depolarization and calcium influx, which is believed to contribute to altered PASMC function. Similarly, in rat PASMCs, bone morphogenetic protein 4 (BMP4) increases Nox4 expression and the resultant ROS drives increased transient receptor potential channel 1 (TRPC1) and TRPC6 expression, leading to elevated basal calcium levels and store-operated calcium entry, followed by increased proliferation [63]. Finally, mammalian target of rapamycin 2 (mTORC2) is a critical regulator of PASMC proliferation, via downregulation of AMP-activated protein kinase (AMPK) and subsequent mTORC1 activation, as well as PASMC survival, via downregulation of AMPK and the pro-apoptotic protein Bim [43]. In PH PASMCs, increased Nox4 expression is required for mTORC2 activation [43], placing Nox4 upstream of another central coordinator of the PASMC function.

Despite this large body of work placing Nox4 at the center of several pathways believed to be critical to reprogrammed PASMC function in PH, attempts at Nox4 inhibition in vivo have produced mixed results. Pharmacologic targeting of Nox has significantly improved over time. Historically, apocynin was used as a Nox inhibitor, but it has since been shown to function primarily as an antioxidant (i.e., ROS scavenger) in vascular cells [64]. Subsequently, novel small molecules that preferentially inhibit Nox1 and Nox4 as well as biological compounds directed against specific Nox isoforms have been developed; their specificity for Nox isoforms and lack of ROS scavenging activity have been characterized with variable rigor [65]. In chronically hypoxic mice, GKT137831, a well-characterized pharmacologic inhibitor of both Nox4 and Nox1, attenuates increased right ventricular hypertrophy and the thickness of the pulmonary vascular wall, but has no effect on right ventricular systolic pressure or muscularization of small arteries [57]. In the MCT-exposed rat, pharmacologic Nox4 inhibition is effective at preventing increases in right ventricular hypertrophy and systolic pressure, and measures of pulmonary arterial stiffness [27]. When given after PH has developed in MCT-exposed rats, Nox4 inhibitors attenuate further progression of disease, but do not reverse it. In contrast, studies evaluating mice with a genetic deficiency of Nox4 find limited protection from CH. In one, neither global constitutive nor global inducible Nox4 deficiency had any effect on right ventricular hypertrophy and systolic pressure, or pulmonary vascular remodeling [66]. In this study, Nox4 deficiency also had no effect on hypoxic pulmonary vasoconstriction in isolated, buffer-perfused lungs. While there was no assessment of changes in the expression of other Nox isoforms in these mice, the use of inducible Nox4-deficient mice eliminates the problem of chronic compensatory mechanisms that could arise in the constitutive Nox4-deficient mice. In the other study, utilizing female mice, global constitutive loss of Nox4 attenuated the elevation in right ventricular systolic pressure, but had no effect on right ventricular hypertrophy or pulmonary vascular remodeling [28]. Potential compensatory changes in other Nox isoforms were not evaluated. Thus, at least in mice, Nox4 does not appear to be necessary for CH-induced pulmonary vascular remodeling or acute hypoxic pulmonary vasoconstriction, while the rat data suggest a potentially more important role in other species, although studies involving pharmacologic inhibitors may suffer from a lack of Nox4-specificity.

#### 2.1.4. Nox3

Little is known regarding any role for Nox3 in the pulmonary vasculature or in PASMCs more specifically. Nox3 mRNA expression was not detected in whole lungs from either normal or MCT rats [67]. However, others have since identified Nox3 in mouse lungs and PAECs [68]. A single nucleotide polymorphism (SNP) in the *Nox3* gene has been found to be associated with PH susceptibility in a Chinese population [69]. A clearer role for Nox3 in pulmonary vascular function awaits further study.

#### 2.1.5. Nox5

Nox5 is expressed in the media and adventitia of pulmonary arteries from PH patients [70]. To our knowledge, however, it is unknown whether Nox5 expression is altered in PH or whether Nox5 contributes to PASMC function.

## 3. Conclusions

Based on the available evidence, it seems clear that Nox plays an important role in modulating PASMC function during the development of PH. The factors regulating Nox expression and activity, along with the downstream effector pathways controlling proliferation, are still being explored, but the data to date yield a complex picture with a built-in redundancy and amplification mechanisms. As detailed exploration of the role of Noxs in controlling PASMCs proliferation continues, there is little doubt that additional pathways are likely to be identified. While Nox inhibition is currently being studied in other diseases, like IPF [71], it remains to be resolved whether Nox can be a useful therapeutic target in PAH. Due to their key roles as modulators of signal transduction throughout the body during normal physiology, and considering adverse findings in animals with genetic deletion of Noxs, it is not unreasonable to suspect that the inhibition of Noxs could bring on unexpected or undesirable outcomes. However, recently developed compounds specifically targeting Nox isoforms to reduce, but not eliminate, ROS production may have better safety profiles for use in vivo [72]. Given that the relative contribution of various Nox isoforms to PASMC hyperproliferation may vary with species, inciting causes of PH and sex, should these compounds ultimately be found safe for use in humans, more data will also be required to ultimately determine exactly which patients might benefit.

## Figures and Tables

**Table 1:** Clinical classification of pulmonary hypertension *.

**Group 1—Pulmonary Arterial Hypertension (PAH)**
Idiopathic
Heritable
Drug and toxin induced
Associated with connective tissue disease; infections; portal hypertension; congenital heart diseases
Long-term responders to Ca^2+^ channel blockers Overt pulmonary venous and/or capillary involvement Persistent pulmonary hypertension of the newborn
**Group 2—Pulmonary Hypertension Due to Left Heart Disease**
Failure with preserved ejection fraction Failure with reduced ejection fraction Valvular disease Congenital/acquired left heart inflow/outflow tract obstruction and congenital cardiomyopathies
**Group 3—Pulmonary Hypertension Due to Lung Diseases and/or Hypoxia**
Chronic obstructive pulmonary disease Restrictive lung disease Other pulmonary diseases with mixed restrictive and obstructive pattern Hypoxia without lung disease Developmental lung disease
**Group 4—Pulmonary Hypertension Due to Pulmonary Artery Obstructions**
Chronic thromboembolic pulmonary hypertension (CTEPH) Other obstructions
**Group 5—Pulmonary Hypertension with Unclear/Multifactorial Mechanisms**
Hematologic disorders Systemic/metabolic disorders Complex congenital heart disease Others

*: Modified from [1].
